# ﻿A new species of *Chrysotus* Meigen (Diptera, Dolichopodidae) from soybean fields in South Dakota, USA

**DOI:** 10.3897/zookeys.1135.95026

**Published:** 2022-12-14

**Authors:** Justin B. Runyon, Eric Beckendorf, Louis S. Hesler

**Affiliations:** 1 Rocky Mountain Research Station, USDA Forest Service, 1648 S. 7th Avenue, Bozeman, Montana 59717, USA Rocky Mountain Research Station, USDA Forest Service Bozeman United States of America; 2 North Central Agricultural Research Laboratory, USDA Agricultural Research Service, 2923 Medary Avenue, Brookings, South Dakota 57006, USA North Central Agricultural Research Laboratory, USDA Agricultural Research Service Brookings United States of America

**Keywords:** Diaphorinae, egg, natural enemy, Nearctic, new species

## Abstract

A new long-legged fly species, *Chrysotussoya***sp. nov.** (Diptera: Dolichopodidae), is described and illustrated from specimens collected in soybean fields near Brookings, South Dakota, USA. The abundance of this species in soybeans suggests it plays an important role as a beneficial predator.

## ﻿Introduction

The genus *Chrysotus* Meigen (Diptera: Dolichopodidae: Diaphorinae) has a worldwide distribution with about 300 described species (Yang et al. 2006), of which 111 species are known to occur in America north of Mexico (Pollet et al. 2004; Runyon and Capellari 2018). Adults of *Chrysotus* species are found in a variety of habitats, but frequently on foliage of low plants and on humid or moist soils. Larvae are poorly known, but likely occur in mud, damp soil, or leaf litter (Pollet and Brooks 2008). Like most other Dolichopodidae, adults and larvae of *Chrysotus* are generalist predators that feed on other invertebrates including aphids, leafhoppers, and thrips (Rathman et al. 1988; Ulrich 2004). Dolichopodidae are often abundant in agroecosystems, and the beneficial role they play as natural enemies of crop pests is increasingly appreciated (Kautz and Gardiner 2019; Harterreiten-Souza et al. 2020; Krey et al. 2020).

In 2016 and 2017, we collected large numbers of an undescribed species of *Chrysotus* on red sticky traps during a study comparing pest and beneficial insect levels between soybean pest management systems in eastern South Dakota, USA (L. Hesler and E. Beckendorf, unpublished data). However, we were unable to remove these specimens from the sticky cards in satisfactory condition for description. In 2021, we re-sampled these soybean fields using red pan traps to obtain suitable specimens, which are described herein. Two specimens of this species were also found in eastern Montana, collected in colored pan traps as part of the ongoing inventory of wild bees in Montana. The purpose of this paper is to describe this new species of *Chrysotus* and provide a name so that it may be used in forthcoming publications on crop management effects on natural enemies in soybeans.

## ﻿Materials and methods

This study is based on specimens collected in 2021 using red pan traps filled with a 50/50 propylene glycol/water mixture and placed in soybean fields at the USDA Agricultural Research Service (ARS), North Central Agricultural Research Laboratory (NCARL) in Brookings, South Dakota, USA. Two additional specimens were collected in 2019 and 2020 in eastern Montana using yellow, white, and blue pan traps deployed for the ongoing inventory of wild bees of Montana at Montana State University. Specimens were transferred to 70% ethanol and dry mounted on pins using hexamethyldisilixane (HMDS) (Brown 1993). Material from this work is housed in the following institutions: Montana Entomology Collection (MTEC), Montana State University, Bozeman, USA; National Museum of Natural History, Smithsonian Institution (USNM), Washington, D.C., USA; U.S. Department of Agriculture, Agricultural Research Service, North Central Agricultural Research Laboratory (NCARL), Brookings, South Dakota, USA.

Label data for the primary type are cited verbatim. Labels are listed from the top label down with data from each label in quotation marks and separated by a semicolon. Lines of text on labels are delimited by a slash (/) and annotations are placed in square brackets (i.e., []).

The morphological nomenclature follows Cumming and Wood (2017). Homologies of the male and female terminalia follow Capellari (2018). To examine male and female terminalia using a compound microscope, specimens were macerated in 85% lactic acid by heating in a microwave oven for three to four 20-second intervals, prior to being transferred to glycerin on a depression slide for illustration. The male postabdomen on intact specimens is rotated approximately 180° and lateroflexed to the right, but in descriptions “dorsal” and “ventral” refer to the true morphological positions prior to genitalic rotation and flexion (e.g., in lateral view, top of the page is ventral while the bottom is dorsal). Measurements of the leg segments are representative ratios given according to the formula: tibia, tarsomeres 1, 2, 3, 4, and 5.

## ﻿Results

### ﻿Family Dolichopodidae, Latreille, 1809

**Subfamily Diaphorinae Schiner, 1864**

#### Genus *Chrysotus* Meigen, 1824

##### ﻿ Chrysotus soya

Taxon classificationAnimaliaDipteraDolichopodidae

Runyon sp. nov.

FD262334-806C-51BF-92ED-0FF2E6174DF7

https://zoobank.org/6D62898C-990C-4547-B3EA-9D8835489A15

Figs 1–3 , 4 , 5, 6 , 7–8

###### Type material.

***Holotype***, ♂ labelled: “South Dakota: Brookings Co/ North Central Ag[ricultural] Res.[earch] Lab/ soybean, 44.3388°, -96.7924°/ 11 Aug 2021, red pan traps/ L. Hesler & E. Beckendorf”; “HOLOTYPE/ ♂ *Chrysotus*/ *soya*/ Runyon [red label]” (USNM, type number USNMENT01828501). ***Paratypes*: South Dakota**: 4 ♂, 57 ♀, same data as holotype (USNM, MTEC, NCARL). **Montana**: 1 ♂, Dawson Co., 1.5 km southwest of Glendive, 47.093354°N, 104.72388°W, 625 m, 10 July 2019, bee bowls, J. Brower (MTEC); 1 ♀, Sheridan Co., N Westby Road, 48.88642°N, 104.06082°W, 644 m, 29–30 August 2020, bee bowls, Z. Pritchard & J. Botti (MTEC).

**Figures 1–3.**
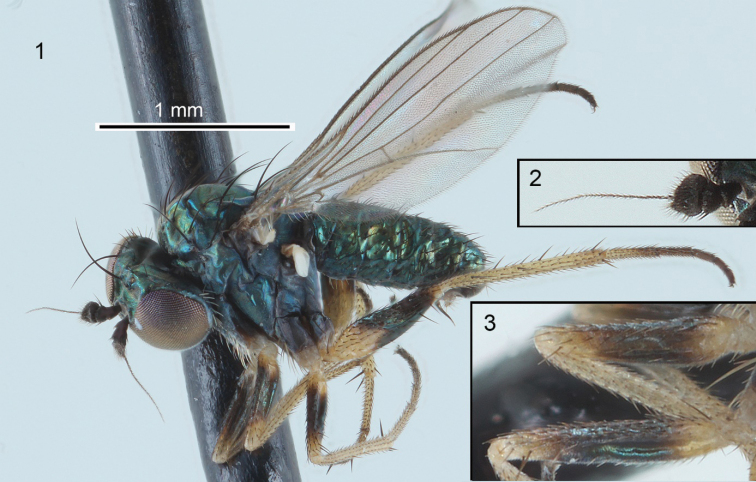
Habitus, antenna, and fore and mid femora of the male holotype of *Chrysotussoya* sp. nov. **1** habitus, left lateral view **2** antenna, medial view **3** fore (top) and mid (bottom) femora, posterior view.

**Figure 4.**
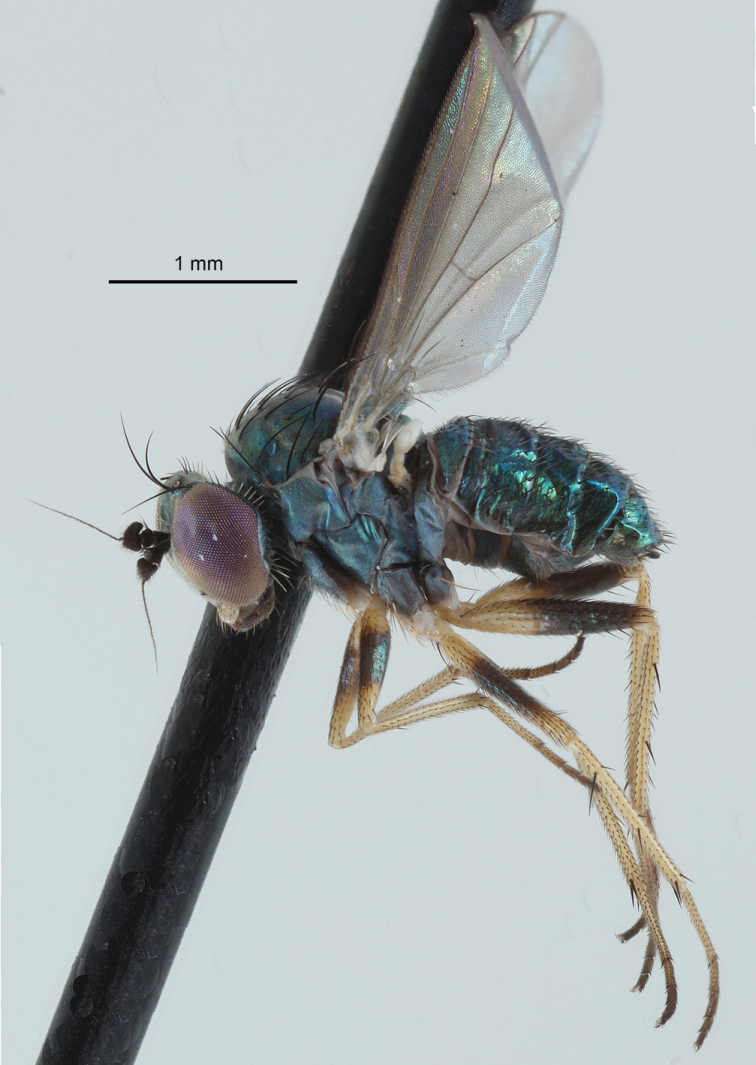
Female habitus of *Chrysotussoya* sp. nov. In some specimens the dark coloration on the fore and mid femora is reduced, and in a few specimens, the fore and mid femora are entirely yellow.

**Figures 5, 6.**
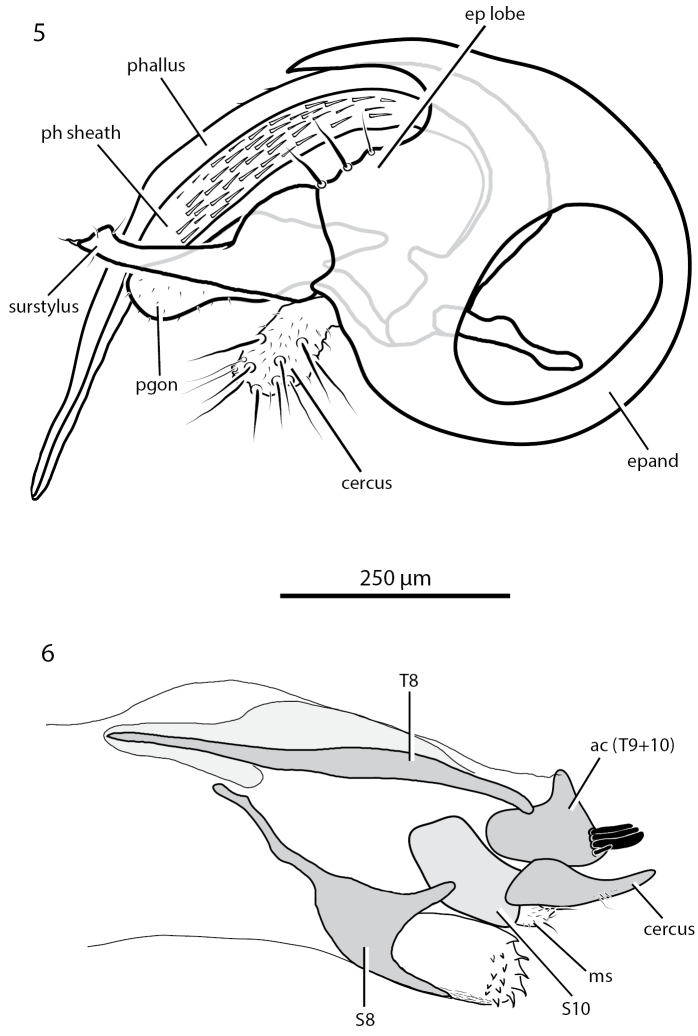
Male and female terminalia of *Chrysotussoya* sp. nov., left lateral view **5** hypopygium of male **6** ovipositor of female. Abbreviations: ac – acanthophorite; epand – epandrium; ep lobe – epandrial lobe; ms – membranous swelling; pgon – postgonite; ph sheath – sheath of phallus; s – sternite; t – tergite.

###### Diagnosis.

Males of *Chrysotussoya* sp. nov. can be distinguished from other known New World species of *Chrysotus* by the following combination of characters: eyes contiguous below antenna; antenna entirely black (Fig. 2); palpus yellow, small, oval; calypteral setae and halteres white; legs lacking male secondary sexual characteristics; and the distinct coloration of the femora (dark brown, except base and apex of fore and mid femora and nearly basal half of hind femora yellow; Figs 1, 3) with tibiae entirely yellow. In Van Duzee (1924), *C.soya* sp. nov. fits in the *C.choricus* species group and keys to *C.parvicornis* Van Duzee (= *C.exiguus* Van Duzee), but the latter species has dark femora with only the extreme tips yellow. In Robinson (1964), *C.soya* sp. nov. keys to *C.flavicauda* Van Duzee, but this species differs in having black calypteral setae and a yellowish hypopygium.

###### Description.

**Male** (Figs 1–3, 5): body length 1.9–2.0 mm; wing length 1.7–1.8 mm. ***Head***: eyes contiguous below, with anterior ommatidia slightly enlarged; upper face narrowly triangular, metallic green with dense light brown pruinosity along eye margins. Frons metallic green with bronze reflections and sparse light brown pruinosity. Postcranium metallic green with stronger bronze reflections and brown pruinosity. Palpus small, oval, light yellow, with 2–3 yellow to light brown setae. Proboscis small, brown with fine brown hairs along margin. Antenna (Fig. 2) entirely black; scape short, funnel-shaped; pedicel subequal in length to scape, spheroidal with subapical circlet of setulae; postpedicel reniform, 1.5 × wider than long, with a small point below insertion of arista-like stylus; arista-like stylus inserted just lateral to apex, length about 2× combined length of scape, pedicel and postpedicel. Postocular setae white with uppermost setae grading to dark brown. ***Thorax***: scutum and scutellum metallic green-blue with strong bronze reflections and sparse light brown pruinosity; about 6 pairs of biseriate acrostichal setae in offset rows; 6 pairs of dorsocentral setae, anterior-most pair small; scutellum with 1 pair of large marginal setae and 1 pair of small lateral setae. Pleuron metallic bluish green with dense gray pruinosity; 2–3 pale setae on lower proepisternum. ***Legs*** (Figs 1, 3): coxae concolorous with pleuron but often slightly browner, with yellow apices; fore coxa with white to yellow-white setae; setae of mid coxa usually white but light brown in one specimen; hind coxa bare except for white to light brown anterodorsal seta. Trochanters yellow. Fore and mid femora yellow at base and apex, with broad dark brown band with green-blue reflections around middle; hind femur yellow with approximately apical half dark brown with green-blue reflections, dark coloration extending further towards base dorsally and ventrally; hind femur with 2–3 distinct anteroventral setae near tip. Tibiae entirely yellow; fore tibia with small anterodorsal seta near 1/4; mid tibia with large anterodorsal seta near 1/4 and 1/2, 2–3 indistinct posterodorsal setae on basal half, and apical ring of 4 setae with ventral seta strongest; hind tibia with anterodorsal seta near 1/4 and 1/2, 4–5 smaller posterodorsal setae, and apical ring of 4 setae. Tarsi unmodified, yellow with distal tarsomeres becoming brown, all legs with small claws and very small pulvilli. Ratios of tibia:tarsomeres for foreleg: 20–10–5–4–3–4; for midleg: 26–14–6–5–4–4; for hindleg: 24–8–6–4–3–2. ***Wing*** (Fig. 1): hyaline, narrowly elliptical, without anal lobe. Vein R_2+3_ nearly straight and slightly but evenly diverging from R_4+5_. Veins R_4+5_ and M_1_ slightly diverging before to just beyond crossvein dm-cu, then nearly parallel in apical fourth of wing. Vein M_1_ ending before wing apex. Crossvein dm-cu placed near 2/5 of wing length, about one-fifth as long as last part of CuA_1_. Calypter white with white setae. Halter stem yellow, knob white. ***Abdomen***: cylindrical, only slightly tapering to apex. Tergites dark metallic green-blue with bronze reflections and sparse gray pruinosity, with dark brown to black setae; tergite 6 with numerous setae. Sternites concolorous with tergites; basal sternites with whitish setae, distal sternites with brown to black setae; sternite 8 covering hypopygial foramen, with small setae. Hypopygium (Fig. 5) partially embedded in tip of abdomen. Hypopygial foramen left lateral. Epandrium dark brown, ventral two-thirds shiny, dorsal one-third covered with minute pale setulae; ventroapical epandrial lobe rather flattened, with 3 broad lobes each bearing a pale seta. Surstylus shining brown, broadened ventrally near base; in lateral view, narrowed apically with slightly broadened and shallowly bilobed apex; in ventral view, spatulate; with apical small pale seta and 2–3 smaller subapical setulae. Cercus pale brown with numerous pale hairs and setae. Phallus narrow with sharply pointed apex; encircled by external membranous sheath that is expanded dorsally, this expanded area contains numerous narrowly-triangular spicules. Postgonites rather large, broadly rounded and covered with sparse microtrichia apically.

**Figures 7, 8.**
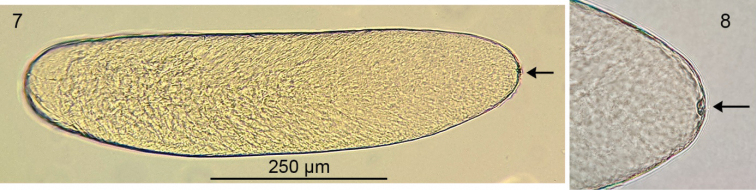
Egg extracted from abdomen of female *Chrysotussoya* sp. nov. **7** egg, lateral view **8** anterior tip of egg. Arrows indicate the micropyle.

**Female** (Figs 4, 6): body length 2.2–2.8 mm, wing length 2.0–2.5 mm. Similar to male except as follows: ***Head***: face wide (width subequal to width of postpedicel), nearly parallel-sided, covered with dense light gray pruinosity which becomes yellowish along eyes. Palpi larger, yellow with base brownish, covered with yellow microtrichia and about five yellow to light brown setae. Postpedicel slightly shorter and wider (2.0× wider than long). ***Legs***: fore and mid femora with dark brown banding often reduced to nearly absent (in a few specimens, the fore and mid femora are entirely yellow). ***Abdomen***: broader, more abruptly tapering to apex. Terminalia (Fig. 6) typical for *Chrysotus* species (Capellari 2018). Acanthophorites (syntergites 9+10) each with four spines. ***Eggs*** (Figs 7, 8): one dissected female contained seven eggs within the abdomen. These eggs were light yellow in color, elongate oval in shape, and of uniform size: approximate length 650 µm, width 170 µm. Chorion smooth and shiny. Yolk homogenous and evenly distributed throughout the cytoplasm. Micropyle located at the anterior tip. No other structures (e.g., aeropyles, nucleus) could be detected using light microscopy.

###### Etymology.

The specific epithet *soya* is derived from soya bean, a common name of soybean, *Glycinemax*, and should be regarded as a noun in apposition.

###### Distribution.

Montana, South Dakota.

###### Remarks.

A heavily female-biased sex ratio of approximately 10:1 (females:males) was found in specimens of *Chrysotussoya* sp. nov. collected in South Dakota soybean fields. This skewed sex ratio occurred in both sets of specimens used in this study, those collected in red pan traps in 2021 (57 females, 5 males), and those collected on red sticky traps in 2016–2017 (1978 females, 218 males). Whether this reflects the true sex ratio is unclear, as females could be more attracted to red color than males.

## ﻿Discussion

*Chrysotussoya* sp. nov. was abundant in soybean fields in eastern South Dakota, and in fact, was the most abundant predatory insect found in these fields (L. Hesler and E. Beckendorf, unpublished data). Because *Chrysotus* species are generalist predators of other arthropods, this suggests that *C.soya* sp. nov. could play a role in pest management. For example, aphids are a frequently reported prey of adult Dolichopodidae (Ulrich 2004) and could contribute to the management of the soybean aphid (*Aphisglycines* Matsumura), the most important arthropod pest of soybeans in North America (Ragsdale et al. 2011). It is estimated that some adult Dolichopodidae could consume one aphid per minute (Rathman et al. 1988) and have the capacity to reduce aphid populations by 50% in wheat fields in Brazil (Bortolotto et al. 2022). Moreover, adult dolichopodids are reported to prey on other insects of economic importance, including caterpillars, leafhoppers, leaf miners, and thrips (Ulrich 2004).

The specimens collected in Montana offer insight into the native habitat of *C.soya* sp. nov. Both Montana specimens were collected in prairie habitat, close to a pond or river. This species is uncommon in Montana – just two specimens were found despite intensive sampling (using yellow, blue, and white pan traps) throughout Montana from 2019–2021. This suggests that *C.soya* sp. nov. naturally occurs in low abundance in grassland habitat in the Northern Great Plains and is able to thrive in the conditions created in at least some soybean fields.

The occurrence of large numbers of *C.soya* sp. nov. in soybean fields provides an opportunity to learn more about this species, and the biology of Dolichopodidae, in general. Of particular interest is documenting what adults are feeding on, and assessing the role they might play in controlling pest species. Despite being widely abundant in agriculture, little is understood about the likely benefits Dolichopodidae provide to growers (Kautz and Gardiner 2019). *Chrysotussoya* sp. nov. could serve as a model to fill this knowledge gap, increase appreciation of dolichopodids, and promote their conservation. Determining the distribution and abundance of *C.soya* sp. nov. in soybeans (and other crops) across the midwestern U.S. and Canada is of clear importance. Lastly, can the larvae be found (likely in soil), and what do they feed on? It is possible that soybean fields are too disturbed for larvae which could develop in soils of nearby native or fallow vegetation with adults emerging and moving into fields to feed and mate. Surprisingly little is known about the larvae of most Dolichopodidae, and the discovery and study of larvae of *C.soya* could contribute significantly to our understanding of the biology of these flies.

## Supplementary Material

XML Treatment for Chrysotus soya
